# A simple score to predict early severe infections in patients with newly diagnosed multiple myeloma

**DOI:** 10.1038/s41408-022-00652-2

**Published:** 2022-04-19

**Authors:** Cristina Encinas, José-Ángel Hernandez-Rivas, Albert Oriol, Laura Rosiñol, María-Jesús Blanchard, José-María Bellón, Ramón García-Sanz, Javier de la Rubia, Ana López de la Guía, Ana Jímenez-Ubieto, Isidro Jarque, Belén Iñigo, Victoria Dourdil, Felipe de Arriba, Clara Cuéllar Pérez-Ávila, Yolanda Gonzalez, Miguel-Teodoro Hernández, Joan Bargay, Miguel Granell, Paula Rodríguez-Otero, Maialen Silvent, Carmen Cabrera, Rafael Rios, Adrián Alegre, Mercedes Gironella, Marta-Sonia Gonzalez, Anna Sureda, Antonia Sampol, Enrique M. Ocio, Isabel Krsnik, Antonio García, Aránzazu García-Mateo, Joan-Alfons Soler, Jesús Martín, José-María Arguiñano, María-Victoria Mateos, Joan Bladé, Jesús F. San-Miguel, Juan-José Lahuerta, Joaquín Martínez-López, GEM/PETHEMA (Grupo Español de Mieloma/Programa para el Estudio de la Terapéutica en Hemopatías Malignas) cooperative study group

**Affiliations:** 1 Hospital General Universitario Gregorio Marañón (HGUGM), IiSGM, Madrid, Spain; 2 Hospital Universitario Infanta Leonor, Madrid, Spain; 3 Hospital Universitario Germans Trias i Pujol, Badalona (Barcelona), Barcelona, Spain; 4 Hospital Clinic, CIBERONC, Barcelona, Spain; 5 Hospital Universitario Ramón y Cajal, Madrid, Spain; 6 University Hospital of Salamanca (HUS/IBSAL), CIBERONC and Cancer Research Institute of Salamanca-IBMCC (USAL-CSIC), Salamanca, Spain; 7 Hospital Universitario Doctor Peset, Valencia, Spain; 8 Hospital Universitario la Paz, Madrid, Spain; 9 Hospital Universitario 12 de Octubre, CIBERONC, Madrid, Spain; 10 Hospital Universitario la Fe, CIBERONC, Valencia, Spain; 11 Hospital Clínico San Carlos, Madrid, Spain; 12 Hospital Clínico Universitario “Lozano Blesa”, Zaragoza, IIS Aragón Spain; 13 Hospital J.M. Morales Meseguer, Murcia, Spain; 14 Hospital Universitario Dr. Josep Trueta, Girona, Spain; 15 Hospital Universitario de Canarias, San Cristóbal de La Laguna, Spain; 16 Hospital Son Llatzer, Palma de Mallorca, Spain; 17 Hospital de la Santa Creu i Sant Pau, Barcelona, Spain; 18 Clínica Universidad de Navarra, CIMA, IDISNA; CIBERONC, Pamplona, Spain; 19 Hospital Donostia, San Sebastián, Spain; 20 Complejo Hospitalario de Cáceres, Cáceres, Spain; 21 Hospital Universitario Virgen de las Nieves, Granada, Spain; 22 Hospital Universitario de la Princesa y Hospital Universitario Quirónsalud, Madrid, Spain; 23 Hospital Vall D’Hebron, Barcelona, Spain; 24 Hospital Universitario de Santiago, Santiago de Compostela, Spain; 25 ICO-L’Hospitalet, IDIBELL, Universitat de Barcelona, Barcelona, Spain; 26 Hospital Universitario Son Espases, Palma de Mallorca, Spain; 27 Hospital Universitario Marqués de Valdecilla, (IDIVAL). Universidad de Cantabria, Santander, Spain; 28 Hospital Universitario Puerta de Hierro Majadahonda, Madrid, Spain; 29 Hospital Universitario Arnau de Vilanova, Lleida, Spain; 30 Hospital General de Segovia, Segovia, Spain; 31 Hospital Parc Taulí, Sabadell (Barcelona), Barcelona, Spain; 32 Complejo Hospitalario Regional Virgen del Rocío, CIBERONC, Sevilla, Spain; 33 Complejo Hospitalario de Navarra, Pamplona, Spain

**Keywords:** Preventive medicine, Risk factors

## Abstract

Infections remain a common complication in patients with multiple myeloma (MM) and are associated with morbidity and mortality. A risk score to predict the probability of early severe infection could help to identify the patients that would benefit from preventive measures. We undertook a post hoc analysis of infections in four clinical trials from the Spanish Myeloma Group, involving a total of 1347 patients (847 transplant candidates). Regarding the GEM2010 > 65 trial, antibiotic prophylaxis was mandatory, so we excluded it from the final analysis. The incidence of severe infection episodes within the first 6 months was 13.8%, and majority of the patients experiencing the first episode before 4 months (11.1%). 1.2% of patients died because of infections within the first 6 months (1% before 4 months). Variables associated with increased risk of severe infection in the first 4 months included serum albumin ≤30 g/L, ECOG > 1, male sex, and non-IgA type MM. A simple risk score with these variables facilitated the identification of three risk groups with different probabilities of severe infection within the first 4 months: low-risk (score 0–2) 8.2%; intermediate-risk (score 3) 19.2%; and high-risk (score 4) 28.3%. Patients with intermediate/high risk could be candidates for prophylactic antibiotic therapies.

## Introduction

Multiple myeloma (MM) is a malignant proliferation of mature clonal plasma cells, which are responsible for the secretion of an excess of clonal immunoglobulins. The disease is commonly associated with the suppression of normal immunoglobulins (Immunoparesis), leading to severely impaired humoral immunity; in addition, patients with MM also show dysfunctional cellular and innate immunity [1]. This complex failure of immunosurveillance mechanisms also leaves patients highly vulnerable to viral (10-fold increase) and bacterial (7-fold increase) infections [2, 3], which are a significant cause of morbidity and mortality in MM, particularly within the first months of therapy [4–6]. Early mortality is related to myeloma progression, especially in elderly patients [7] and infections can additionally increase the risk of early-related myeloma mortality because of the delay or adjustment of doses.

Recent advances in treatments have improved the survival of patients with MM [8–10]. However, myeloma treatments, including new biological agents, might also impact the immune system, either positively or negatively, increasing the risk of infection [11–15]. The most common side-effect in this context is cytopenia, which is associated with increased susceptibility to bacterial and viral infections, particularly when a high dose of chemotherapy followed by autologous stem cell transplant is administered [12]. Corticosteroids are included in most drug combinations for MM, but they have well-established immunosuppressive effects [6]. Similarly, proteasome inhibitors (PIs) induce T-cell dysfunction and are associated with an increased risk for varicella-zoster virus (VZV) reactivation [16, 17]. Immunomodulatory drugs (IMiDs) may have a protective effect by enhancing natural killer (NK) and T-cell function, but they are associated with cytopenia, and there is evidence that their use is accompanied by an increase in serious infections [11, 18–21]. Finally, CD38-targeting monoclonal antibodies (CD38 MAbs) reduce the number of NK and immunosuppressive regulatory T-cells and are associated with a higher number of infections (e.g., increased risk of VZV infection, and a higher risk of hepatitis B virus reactivation) [22, 23].

The incidence of severe infection in patients with MM seems to be higher during the first months after the diagnosis [2, 4, 5, 24]. Several reports have indicated that the administration of prophylactic antibiotics or the use of vaccination protocols before beginning myeloma treatment reduces the frequency and severity of early infections, but their use remains controversial [25–28]. Recent results of the TEAMM study have shown that the addition of prophylactic levofloxacin to active myeloma treatment during the first 12 weeks of therapy significantly reduces febrile episodes and deaths versus placebo [29, 30].

Risk scores to predict the probability of infection could help in identifying patients at higher risk of infection who may benefit from individualized prophylactic antibiotic treatments. Along this line, a scoring system was developed in a sub-study of the phase 3 FIRST clinical trial [31], which characterized treatment-emergent infections in transplant-ineligible elderly patients treated with lenalidomide or thalidomide. Results showed that Eastern Cooperative Group Oncology (ECOG) performance status [32] ≥ 2, β2microglobulin (B2M) ≥ 6 mg/L, lactate dehydrogenase >200 U/L, and hemoglobin <11 g/dL were significantly associated with a high risk of infections [24]. Based on this model, the benefit of adding prophylactic antibiotics was useful only in a subset of patients with a high risk of infections. While the risk score was externally validated in three independent data sets (MM-003, MM-009, and MM-010^24^), the results have not been confirmed in other series and, particularly, in the transplant-candidate setting.

The present study aimed to analyze the rate of severe infection in a large series of newly diagnosed transplant-eligible and -ineligible patients with MM treated with novel agents in the context of prospective clinical trials of the Spanish Myeloma Group (PETHEMA, *Grupo Español de Mieloma*, GEM). Furthermore, we aimed to identify the clinical and biological variables associated with the risk of early severe infection that could be used to generate a score for identifying patients at higher risk of early severe infection in all subsets of MM in the era of new agents.

## Patients and methods

### Study design

The present study was designed to develop a scoring system to predict the risk of early severe infection in patients with MM. For this purpose, we analyzed all infectious events in a large series of transplant-eligible and -ineligible patients with newly diagnosed MM treated in four GEM clinical trials: GEM2005 > 65, GEM2010 > 65, GEM2005 < 65, and GEM2012 < 65. The protocols of these studies are included as Tables S1 and S2 in Supplementary Material [33–36]. We analyzed infectious events focusing on the first 6 months in an attempt to harmonize the population of patients analyzed and avoid post-transplant period bias of the GEM2005 < 65 and GEM2012 < 65 trial, where the autologous transplant was performed after 6 cycles of induction. Regarding the GEM2010 > 65 trial, antibiotic prophylaxis was mandatory during the first 3 months (oral levofloxacin, 500 mg daily), and therefore, we elected to exclude it from the final analysis, although it did allow us to compare the incidence of early severe infection with GEM2005 > 65 where prophylaxis was not mandatory, with both trials in transplant-ineligible populations. Infectious events were graded according to the National Cancer Institute–Common Toxicity Criteria for adverse events (NCI-CTCAE), version 4.0. We considered early severe infection as an infection occurring in the first 4 months and when it was grade ≥3 (as is considered by NCI-CTCAE) and, in the case of pneumonia, irrespective of the grade of severity. We also evaluated the demographics, clinical and/or biological variables to test for associations with a higher risk of early severe infection. In addition, we tested the prediction value of previously published scores [24] for infection in patients with MM.

### Patients

A total of 1347 newly diagnosed patients with symptomatic MM who had received at least one treatment dose were analyzed in this post hoc study. The median age of patients was 62 years (range 25–88) and the median follow-up was 81.7 months. Five-hundred patients who were ineligible for autologous transplant and aged ≥65 years were included in trials GEM2005 > 65 [33] and GEM2010 > 65 [34], and 847 patients who were transplant candidates and aged < 65 years were included in trials GEM2005 < 65 [35] and GEM2012 < 65 [36]. All patients were diagnosed according to the International Myeloma Working Group criteria [37] and provided written informed consent before the screening. The trials were registered on ClinicalTrials.gov; data were reviewed by an external clinical research organization and centrally assessed, and the efficacy and safety have been previously reported [33–36]. The main clinical and biological characteristics of patients included in the analysis are summarized in Table 1.

**Table 1:** Main clinical characteristics of the patients.

Variables		Number of patients (%)
Total of patients		1347 (100)
**Protocol**	GEM05 > 65	259 (19.2)
	GEM05 < 65	389 (28.9)
	GEM2010 > 65	241 (17.9)
	GEM2012 < 65	458 (34.0)
**Sex**	Male	703 (52.2)
	Female	644 (47.8)
**Age (years)**	>75	168 (12.5)
	≤75	1179 (87.5)
	>55	771 (57.2)
	≤55	576 (42.8)
**ECOG PS**	0–1	788 (58.5)
	>1	559 (41.5)
**Hb (g/dL)**	≤11	760 (56.4)
	>11	587 (43.6)
**ISS Stage**	1–2	997 (74.0)
	3	350 (26.0)
**Monoclonal Component (g/L)**	≤40	509 (37.8)
	>40	838 (62.2)
**Bone lytic lesion**	Yes	1160 (86.1)
**Extramedullary disease**	Yes	237 (17.6)
**Score FIRST**	High risk (2 to 5)	465 (34.5)
	Low risk (-3 to 1)	882 (65.5)
**LDH**	Normal	1142 (84.8)
**High-risk cytogenetic**	Yes	240 (20.2)
**abnormalities**	NA	157
**Albumin (g/L)**	≤30	268 (19.9)
**Type MM**	Non-IgA	991 (73.6)
	IgA	356 (26.4)

ECOG PS Eastern Cooperative Oncology Group Performance Status, Hb Hemoglobin, NA not available,

β2 M β2microglobulin, ISS Stage International Staging System Stage, LDH lactate dehydrogenase, IgA Immunoglobulin A, MM Multiple Myeloma.

Antibiotic prophylaxis was not mandatory in GEM2005 < 65, GEM2005 > 65 or GEM2012 < 65 (antibiotics were administered at the discretion of the trial center), whereas in GEM2010 > 65 a prophylactic antibiotic was used as per protocol during the first 3 months.

### Outcomes and variables analyzed

The primary outcomes were the early severe infection incidence and the clinical and biological variables associated with an increased risk of severe infection, which were used to build a score to predict the risk of early severe infection. Secondary outcomes included (1) to identify the number of patients with at least one infection in the series, focusing on the first 6 months, and the number, severity, and type of infectious events in the global series and in each of the clinical trials; (2) to compare the incidence of early severe infection between the transplant-ineligible populations, GEM2005 > 65, without antibiotic prophylaxis, and GEM2010 > 65, with mandatory antibiotic prophylaxis; and finally (3) to determine the mortality due to infection and the impact of experiencing a severe infection on overall survival (OS).

Most of the continuous variables were dichotomized before their inclusion in the analysis using clinical criteria and the area under the receiver operating characteristic (ROC). The following characteristics were analyzed: age (≤75 vs >75, >55 vs ≤55 years old), sex, ECOG 0–1 vs >1, β2microglobulin (β2 M) in mg/L ( < 5.5 vs >5.5, <6 vs >6, ≤3 vs >3), serum lactate dehydrogenase (normal or higher than the upper limit of the normal range), staging according to Durie–Salmon staging system [37, 38], staging according to the International Staging System (ISS) [37, 39, 40] 1–2 versus 3, high versus standard risk cytogenetic abnormalities [41], monoclonal component (≤40 g/L vs >40 g/L), type of involved paraprotein (IgA vs non-IgA), hemoglobin level (≤11 g/dL vs >11 g/dL), serum albumin level (≤30 g/L vs >30 g/L), risk of early infection using FIRST score [24] [high-risk (2–5 points) vs low risk (−3–1 points)], presence of bone lytic lesions, autologous transplant and presence of extramedullary disease. Platelet level, creatinine, calcium, C-reactive protein (CRP) in blood, and plasma cell infiltration in bone marrow were included as continuous variables. Variables were selected by expert assessment and were evaluated with univariate and multivariate models based on their clinical and/or biological relevance.

### Statistical analysis

All data were entered into the Redcap database [42, 43] (Vanderbilt University, Nashville, TN, USA). Statistical analysis was performed with the SPSS Statistics for Windows program, version 25.0 (IBM Corp., Armonk, NY, USA). Continuous variables are represented by means and standard deviation. For categorical variables, the results are expressed in frequencies and percentages. Normality analysis was performed using graphical tests and the Kolmogorov–Smirnov test.

To compare the differences between two or more groups, parametric tests (Student’s t or ANOVA) or non-parametric tests (Mann–Whitney or Kruskal–Wallis) were used, as necessary. The association between qualitative variables was studied using Pearson’s chi-square test or Fisher’s exact test. Time to infection was measured using cumulative incidence curves considering death without infection as a competitive event. The evolution of cumulative incidence between groups was compared using the Pepe and Mori test.

Univariate and multivariate logistic regression was performed to calculate the odds ratio (OR) for severe infections at 4 months with a 95% confidence interval (CI). In addition, we built a predictive model including variables of clinical relevance and variables that showed a statistical association (*p* < 0.1) in the univariate analysis. Model calibration was performed with the Hosmer–Lemeshow test, and its discrimination was assessed by calculating the area under the ROC curve (AUC). The coefficients of the logistic regression models were converted into a simplified score to facilitate their application in clinical practice, dividing each coefficient by the coefficient with the lowest value and rounding it to an integer. Subsequently, the incidence of severe infection within the first 4 months was estimated based on the score, and the sub-hazard ratio (sHR, competing risk regression) was calculated for the different groups. Two-tailed tests were used, and results with *p* < 0.05 were considered statistically significant.

## Results

### Infectious events

Focusing on the first 6 months, 327/1347 patients (24.3%) experienced at least one infection (227 patients with one infectious event, 74 patients with two, 19 patients with 3, and seven patients with >3 infectious events). 161/1347 patients (12.5%) experienced at least one severe infection (49.2% of all the patients with any-grade infection reported).

**Fig. 1:**
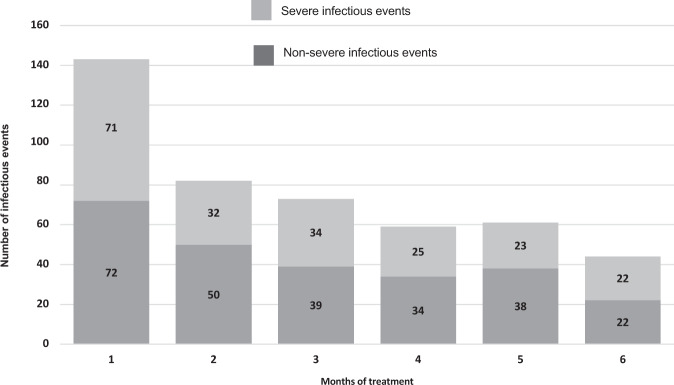
Distribution of the number of infectious events per month from treatment.

Data on the number, severity, and type of infectious events in the global series and by clinical trials are shown in Table 2. Most infectious events were respiratory infections with 273 events (59.1% of total infectious events), including 96 events (21.7%) of pneumonia. There were 40 events (8.7%) considered fever episodes, and 38 events (8.2%) considered urinary tract infections. A total of 13 events (2.8%) were catheter infections, and 11 events (2.4%) were bacteremia episodes. Regarding the infectious pathogens documented during the first 6 months after treatment initiation, results were available in 50 infectious events (10.8% of the total), and 56% of events in which a pathogen was documented were bacterial infections (Table S3 in Supplementary Material). Figure 1 shows the distribution of infectious events per month in this period: 357 (77.3%) of 462 any-grade infectious events and 162 (78.3%) of 207 severe infectious events occurred within the first 4 months of treatment.

**Table 2:** Rate of any-grade and severe infection by clinical trial, by most frequent type of infections in the first 6 months.

Protocol	*N* Events (%)	Severe (%^)	RTIs/Pneumonia (%^)	UTI (%^)	Febrile syndrome (%^)	BSI (%^)
**GEM05** > **65**	**103 (22.3)**	37 (35.9)	57 (55.3)/23 (22.3)	11 (10.7)	14 (13.6)	0 (0)
**GEM05** < **65**	**143 (31.0)**	65 (45.5)	106 (74.1)/36 (25.2)	10 (7.0)	3 (2.1)	5 (3.5)
**GEM10** > 65*	**48 (10.3)**	16 (33.3)	18 (37.5)/5 (17.9)	1 (2.1)	6 (12.5)	0 (0)
**GEM12** < **65**	**168 (36.4)**	89 (53.2)	92 (54.8)/32 (19)	16 (9.5)	17 (10.1)	19 (11.3)
**Total**	**462 (100)**	**207 (44.8**^**¶**^**)**	**273 (59.1**^**¶**^**)/96 (21.7**^**¶**^**)**	**38 (8.2**^**¶**^**)**	**40 (8.7**^**¶**^**)**	**24 (5.2**^**¶**^**)**

*N* events (%): number of any-grade infectious events by protocol and in the global series (percentage of total any-grade infectious events), Severe: number of severe infectious events by protocol and in the global series. RTIs/Pneumonia: number of any-grade respiratory tract infections/Pneumonia by protocol and in the global series; UTI: any-grade urinary tract infections by protocol and in the global series, BSI Bloodstream infections, including bacteremia and catheter infection by protocol and in the global series. (%^) percentage of total infectious events in each protocol.

^¶^Percentage of total any-grade infectious events.

*In GEM10 > 65, antibiotic prophylaxis was mandatory during the first 3 months.

**Fig. 2:**
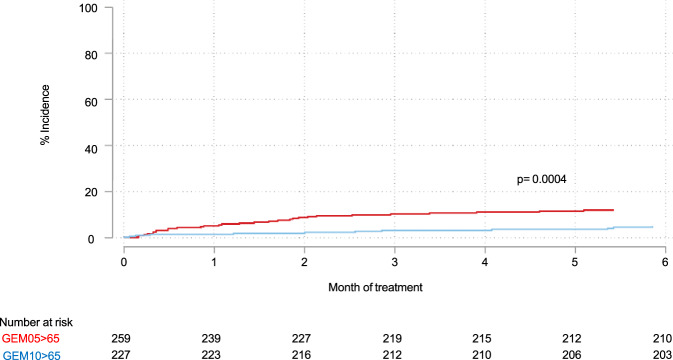
Cumulative incidence of severe infections comparing non-candidate transplant patients.

**Fig. 3:**
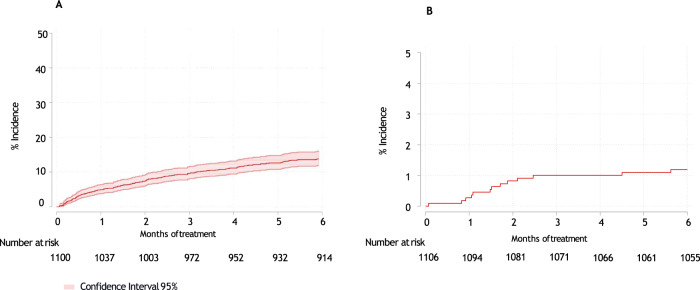
Cumulative incidence. **A** Cumulative incidence of severe infection and **B** mortality by infection.

Comparative analysis of the transplant-ineligible patients showed that the incidence of early severe infection was higher (HR 2.57, *p* < 0.001) in the GEM2005 > 65 trial than in the GEM2010 > 65 trial (Fig. 2), where antibiotic prophylaxis in the first 3 months was mandatory. Accordingly, patients enrolled in the GEM2010 > 65 trial (*n* = 241) were excluded from the risk score final analysis (*n* = 1106) due to the use of antibiotic prophylaxis. The cumulative incidence of serious infections at 6 months was 13.8%, and 11.3% experienced the first episode within the first 4 months (Fig. 3A).

**Fig. 4:**
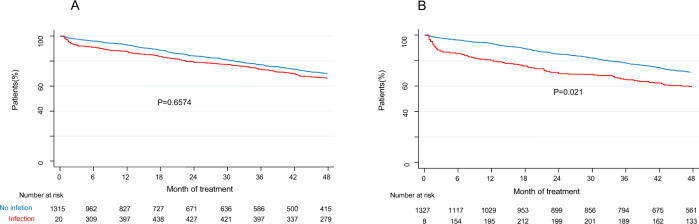
Impact on overall survival of infection. **A** Impact on overall survival of any-grade infection and **B** severe infection.

If we consider the 4 CTs, a total of 59 patients died in the first 6 months of treatment (46 patients in the first 4 months), of them, 16 patients died by infection in the first 6 months of treatment (1.2%) and 13 patients in the first 4 months (1%). If GEM2010 > 65 is not considered, 13 patients died by infection in the first 6 months of treatment and 11 patients in the first 4 months, as is shown in Fig. 3B. Six had respiratory tract infections, of which five were pneumonia. Infection of any grade did not affect OS (*p* = 0.660) (Fig. 4A); however, severe infection had an impact on OS (*p* = 0.021) in our series (Fig. 4B).

### GEM-PETHEMA and FIRST scores

Initially, we tested the reproducibility of the FIRST score [24] in our three clinical trials (GEM2010 > 65 trial was excluded from the final analysis). We found no significant differences in the incidence of early severe infection between the low or high-risk group (11.0% vs. 13.0%, respectively, *p* = 0.347). Similarly, no differences were observed for the group of transplant-eligible (13.3% vs. 13.0% *p* = 0.255) and -ineligible (6.5% vs. 10.5% *p* = 0.50) patients, or when the protocols were analyzed separately.

A total of 126 patients experienced a severe infection in the first 4 months of treatment. Tables 3, 4 show the results of the univariate and multivariate logistic analysis of the variables associated with a higher risk of early severe infection in the first 4 months. Four variables were selected in the multivariate analysis (AUC = 0.64, 95% CI: 0.58–0.68): albumin ≤30 g/L (OR 2.12, *p* < 0.001), ECOG > 1 (OR 1.73, *p* = 0.005), male sex (OR 1.50, *p* = 0.037) and non-IgA MM type (OR 1.49, *p* = 0.091).

**Table 3:** Univariate logistic regression analysis.

Variables	Odds Ratio		*p*-value	95% Confidence interval	Number of patients
**ECOG PS 0–1 vs** > **1**		1.84	0.001	1.26–2.68	1103
**Age** > **vs** ≤ **55**		1.19	0.354	0.82–1.72	1106
**Female vs male**		0.65	0.026	0.44–0.95	1106
**Monoclonal Component** > **vs** ≤ **40** **g/L**		1.42	0.065	0.98–2.05	1106
**Albumin** > **vs** ≤ **30** **g/L**		2.32	<0.001	1.55–3.48	1102
**non-IgA type MM vs IgA**		1.38	0.166	0.87–2.19	1106

ECOG PS Eastern Cooperative Group Oncology Performance Status, IgA Immunoglobulin A, MM Multiple Myeloma.

**Table 4:** Multivariate logistic regression analysis.

Variables	Odds ratio	*p*-value	95% Confidence interval	Weight (points)
**Albumin** ≤ **30** **g/L**	2.12	<0.001	1.40–3.20	1
**ECOG PS** > **1**	1.73	0.005	1.18–2.54	1
**Male sex**	1.50	0.037	1.02–2.20	1
**Non-IgA type MM**	1.49	0.091	0.93–2.39	1

ECOG PS Eastern Cooperative Oncology Group Performance Status, IgA Immunoglobulin A, MM Multiple Myeloma.

**Fig. 5:**
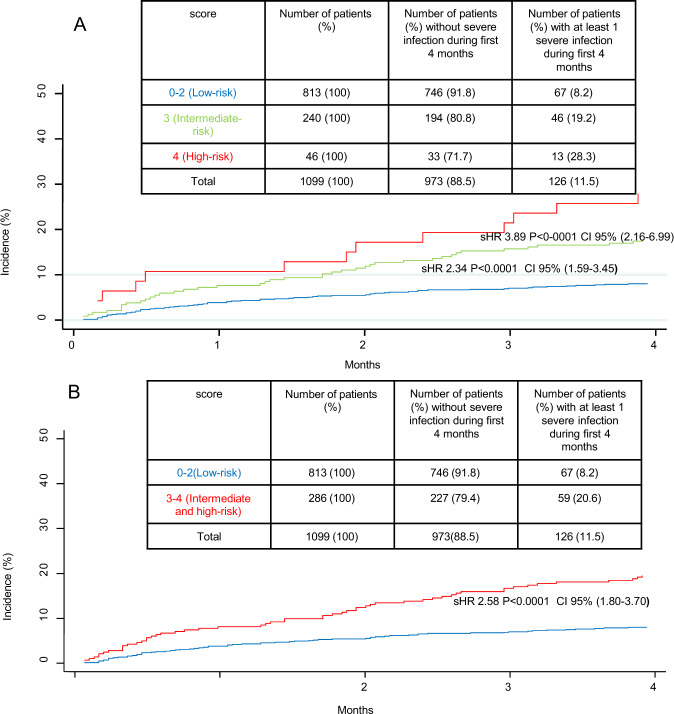
Cumulative incidence of early severe infection according to risk group by Score GEM. **A** Three risk groups with different probabilities of early severe infection. **B** Two risk group when intermediate and high risk were grouped. *sHR* sub-Hazard ratio, CI 95% confidence interval.

We then generated a score, termed GEM-PETHEMA, to predict the risk of infection. The weight of each variable with a higher risk of early severe infection in the multivariate analysis was given 1 point (Table 4). The score facilitated the identification of three risk groups with different probabilities of early severe infection: 8.2% in the low-risk group (0–2 points), 19.2% in the intermediate-risk group (3 points) (sHR 2.34, 95% CI 1.59–3.45, *p* < 0.001) and 28.3% in the high-risk group (4 points) (sHR 3.89, 95%CI, 2.16–6.99 *p* < 0.001) (Fig. 5A). Then, when we compared the low versus intermediate/high-risk groups, the probability of early severe infection was 8.2% and 20.6%, respectively (sHR 2.58, 95% CI 1.80–3.70, *p* < 0.0001) (Fig. 5B). Finally, we tested the GEM-PETHEMA score only in the group of transplant-eligible patients, and the results were reproducible (AUC = 0.62, 95% CI, 0.56–0.67).

## Discussion

In the present study, we sought to evaluate risk factors associated with an early severe infection in a large population of patients with newly diagnosed MM (both young and elderly) treated in clinical trials with therapies based on PIs and IMiDs. Our results show that a relevant number of MM patients, including those treated with novel drugs, develop infections, especially within the first 4 months from the initiation of treatment. In addition, of the total number of infections, almost half of them were considered as serious, which is consistent with the results of other series [2, 4, 24].

Although an improvement in early mortality rate is expected with the introduction of new agents that achieve rapid tumor control as compared with conventional chemotherapy (~5%) [4], our results show that the presence of severe infections still has an impact on the survival of patients.

The cumulative incidence of severe infection after excluding patients enrolled in the GEM2010 > 65 trial (total *n* = 1106) was 13.8% in the first 6 months. Notably, most patients had their first serious infection within 4 months of starting treatment. While the mortality associated with infection in the first 6 months was low (1.2%), and no differences in mortality were observed between GEM2010 > 65 and the other three CTs, it is important to note that most of the deaths occurred in the first 4 months (1%). Even though infection of any degree did not affect OS, severe infection significantly influenced survival.

Of note, about one out of five infections in our analysis involved the lower respiratory tract in the form of pneumonia. Regarding respiratory events by protocol, it is interesting to note that they were lower in patients treated in the GEM2010 > 65 trial [34], where the administration of prophylactic antibiotics was mandatory per protocol during the first 3 months.

In line with our results, a post hoc analysis of the FIRST clinical trial [31] revealed that the number of infections was higher during the first 4 months of therapy. In total, 21% of the patients experienced a grade ≥3 infection in the first 18 months of the therapy, with more than half (56%) occurring in the first 4 months. ECOG status, β2 M, lactate dehydrogenase, and hemoglobin levels are defined high- or low-risk groups, with 24% versus 7% of early severe infection risk, respectively [24]. This score [24] was developed in elderly patients, but there is a lack of data in younger patients. Moreover, when we tested the FIRST score [24] in our series, we found no significant association with a higher risk of early severe infection either in the group of transplant-eligible and -ineligible candidates, and this lack of congruence of our score and that of Dumontet et al. in elderly patients should be answered in future studies.

We searched for a new, simple, and easily applicable score in transplant-eligible and -ineligible candidates that could be used to define groups of patients with a higher risk of acquiring an infection. The combination of low albumin levels (≤30 g/L), performance status according to the ECOG scale (ECOG > 1), male sex, and non-IgA type MM (1 point each) classified the patients into three risk groups, and those at intermediate- and high-risk had a 20% and 30% probability, respectively, of early serious infection as compared with only 8% in those at low-risk. Importantly, the GEM score is valid for both young and elderly populations.

The TEAMM study [29, 30] has shown that the use of prophylactic levofloxacin (500 mg, daily) during the first 4 months in newly treated patients with MM reduced febrile episodes and deaths, and only a low number of mild adverse events were reported, without antibiotic-resistant organisms and healthcare-associated infections [29, 30]. Although this information is of interest, physicians may be concerned about over-treatment with the risk of inducing antibiotic-resistant strains. A strength of our score system is that it could help to individualize the prophylactic antibiotics regimen, for use only in the intermediate- and high-risk group of patients.

Our study has several limitations. First, the results were generated from a post hoc analysis, as the design of the included clinical trials did not consider the incidence of infections as the main endpoint. The patients were selected by the inclusion and exclusion criteria, and it is necessary to reproduce this score in both the real world with patients and clinical trials. Second, the treatment regimens and the characteristics of the patients (eligible vs. ineligible for the hematopoietic transplant) were heterogeneous. Third, we included in the descriptive analysis the patients treated in the GEM2010 > 65 trial, as it was the only trial in which antibiotic prophylaxis was mandatory. Comparative analysis revealed that despite a moderately toxic regimen, particularly for patients >80 years of age, those treated with this scheme had significantly fewer early severe infections than peers belonging to the GEM2005 > 65, also elderly patients, but with non-mandatory antibiotic prophylaxis; however, we elected to exclude the former trial from the final univariate and multivariate analysis to avoid bias because of the prophylactic antibiotics. Fourth, in the three clinical trials used to generate our score, antibiotic prophylaxis was not mandatory and was at the discretion of each participating center and, this information has not been collected in the clinical trials databases, but in general, antibiotic prophylaxis was not performed. Fifth, patients undergoing autologous hematopoietic stem cell transplantation frequently experience infections during the transplant period due to post-chemotherapy aplasia and mucositis, characteristic of conditioning treatments with high doses of melphalan. In the case of the GEM2005 < 65 and GEM2012 < 65 trial, this was performed after 6 cycles of induction, and so only the severe infections occurring in the first 6 months of treatment were considered. Sixth, the potential impact of comorbidities, immunoparesis, and the cumulative dose of corticosteroids on the infection risk were not evaluated. Finally, anti-CD38 monoclonal antibodies (mAbs) were not taken part in the treatment in these trials. It is well known the increased risk of infections with these mAbs, and a score including age, LDH, albumin, and ALT at baseline has been also built to identify patients at higher risk of infections when they receive anti-CD38 mAbs [44, 45]. As the GEM-PETHEMA group is involved in new trials with anti-CD38 mAbs, this score will be evaluated and if new risk factors emerge, the model could be revisited.

The strengths of the study include the multicenter nature of the trials, the inclusion of a large number of patients to establish differences according to the variables analyzed, the prior publication in peer review journals, the continuous updating of the databases, and the results comparable with prior studies related to infections in patients with MM.

In conclusion, our study confirms that a high proportion of serious infections occur within the first 4 months. Male sex, ECOG PS > 1, non-IgA type MM and albumin ≤30 g/L allowed us to discriminate three subgroups of patients with different risk of early severe infection. When the intermediate and high risk were grouped, the differences persist, so patients at intermediate/high risk are the ideal candidates to be treated with prophylactic antibiotics, although this should be validated in independent cohort studies.

## Supplementary information

Table S1

Table S2

Table S3

Suppementary legends

List of investigators in the GEM/PETHEMA
