# Supplementary material for: A simple score to predict early severe infections in patients with newly diagnosed multiple myeloma
Source: Blood Cancer J. 2022 Apr 19;12(4):68. doi: 10.1038/s41408-022-00652-2 (PMC9018751; doi:10.1038/s41408-022-00652-2)
Supplement: Supplementary file 1 — Table S1 [file 41408_2022_652_MOESM1_ESM.docx]

|  | **GEM2005>65**  ([NCT00443235](https://clinicaltrials.gov/ct2/show/NCT00443235)) | **GEM2010>65**  ([NCT01237249](https://clinicaltrials.gov/ct2/show/NCT01237249)) |
| --- | --- | --- |
| **Trial design** | N=260 patients  Randomized 1:1  **Treatment arm A:**  Induction 6 cycles VMP  Maintenance: VP / 3 months for 3 years.  **Treatment arm B:**  Induction: 6 × VTP  Maintenance: VT / 3 months / 3 years | N=240 patients  Randomized 1:1  **Sequential arm:**  9 cycles VMP + 9 cycles Rd  **Alternating arm:**  9 × VMP followed by 9 × Rd  9 × Rd followed by 9 × VMP |
| **Main Inclusion criteria** | - Age 65 years or older* - Symptomatic multiple myeloma - Measurable disease. - No prior myeloma treatment | |

**Table S1: Trial design and main inclusion criteria for the GEM2005>65 and GEM2010>65 trials**

VBMCP: Vincristine, BCNU, Cyclophosphamide, Melphalan, Prednisone. VBAD: Vincristine, BCNU, Adriamycine, Dexamethasone. VRD: Bortezomib, Lenalidomide, Dexamethasone *Median age (GEM2010>65): 75 years, (GEM2005>65): 73 years.
