# Supplementary material for: A simple score to predict early severe infections in patients with newly diagnosed multiple myeloma
Source: Blood Cancer J. 2022 Apr 19;12(4):68. doi: 10.1038/s41408-022-00652-2 (PMC9018751; doi:10.1038/s41408-022-00652-2)
Supplement: Supplementary file 2 — Table S2 [file 41408_2022_652_MOESM2_ESM.docx]

**Table S2: Trial design and main inclusion criteria for the GEM2005<65 and GEM2012<65 trials**

|  | **GEM2005<65**  ([NCT00461747](https://clinicaltrials.gov/ct2/show/NCT00461747)) | **GEM2012<65**  ([NCT01916252](https://clinicaltrials.gov/show/NCT01916252)) |
| --- | --- | --- |
| **Trial design** | N=389 patients  Randomized 1:1:1  **Induction treatment**:  **Group A:**  VBMCP-VBAD+Bortezomib  **Group B:**  Thalidomide+Dexamethasone  **Group C**  Thalidomide+Dexamethasone+Bortezomib  All groups high-dose melphalan-200 (MEL-200)  **Maintenance treatment:**  Interferon (Group M1) or Thalidomide (Group M2) or Thalidomide+Bortezomib (Group M3) during three years. | N=458 patients  Randomized 1:1  **Treatment arm A:**  VRD-GEM induction treatment followed by high-dose melphalan-200 (MEL-200)  **Treatment arm B:**  VRD-GEM induction treatment followed by busulfan-melphalan (BUMEL) |
| **Main Inclusion criteria** | - Patients under 65 years old - Candidate for autologous stem cell transplant - Symptomatic multiple myeloma - Measurable disease. - No prior myeloma treatment | |

VBMCP: Vincristine, BCNU, Cyclophosphamide, Melphalan, Prednisone. VBAD : Vincristine, BCNU, Adriamycine, Dexamethasone. VRD: Bortezomib, Lenalidomide, Dexamethasone.
