# Supplementary material for: A simple score to predict early severe infections in patients with newly diagnosed multiple myeloma
Source: Blood Cancer J. 2022 Apr 19;12(4):68. doi: 10.1038/s41408-022-00652-2 (PMC9018751; doi:10.1038/s41408-022-00652-2)
Supplement: Supplementary file 3 — Table S3 [file 41408_2022_652_MOESM3_ESM.docx]

**Table S3. Infectious pathogens documented during the first 6 Months**

| **Infectious pathogen** | **Number of events** |
| --- | --- |
| **Bacterial**  *S. pneumoniae*  *E. coli*  *S. aureus*  *S. epidermidis*  *Pseudomonas spp*  *Serratia spp*  *Proteus spp*  *Enterobacter spp*  *S. sonnei* | **28**  7  6  4  3  4  1  1  1  1 |
| **Viral**  *Influenza virus*  *H. influenza* | **12**  11  1 |
| **Fungal**  *Aspergillus spp*  *Candida spp*  *P. jirovecii* | **10**  2  7  1 |
| No documented | 412 |
| **Total** | **462** |
