# Supplementary material for: A simple score to predict early severe infections in patients with newly diagnosed multiple myeloma
Source: Blood Cancer J. 2022 Apr 19;12(4):68. doi: 10.1038/s41408-022-00652-2 (PMC9018751; doi:10.1038/s41408-022-00652-2)
Supplement: Supplementary file 4 — Suppementary legends [file 41408_2022_652_MOESM4_ESM.docx]

**Supplementary legends**

**Table S1** (Supplementary material). Trial design and main inclusion criteria for the GEM2005>65 and GEM2010>65 trials.

VMP: Bortezomib, Melphalan, Prednisone. VTP: Bortezomib, Thalidomide, Prednisone. VP: Bortezomib, prednisone. VT: Bortezomib, Thalidomide. Rd: Lenalidomide, dexamethasone.

**Table S2** (Supplementary material). Trial design and main inclusion criteria for the GEM2005<65 and GEM2012<65 trials.

VBMCP: Vincristine, BCNU, Cyclophosphamide, Melphalan, Prednisone. VBAD: Vincristine, BCNU, Adriamycine, Dexamethasone. VRD: Bortezomib, Lenalidomide, Dexamethasone. *Median age (GEM2010>65): 75 years, (GEM2005>65): 73 years.

**Table S3** (Supplementary material). Infectious pathogens documented during the first 6 Months
