# Supplementary material for: A simple score to predict early severe infections in patients with newly diagnosed multiple myeloma
Source: Blood Cancer J. 2022 Apr 19;12(4):68. doi: 10.1038/s41408-022-00652-2 (PMC9018751; doi:10.1038/s41408-022-00652-2)
Supplement: Supplementary file 5 — List of investigators in the GEM/PETHEMA [file 41408_2022_652_MOESM5_ESM.docx]

**List of investigators in the GEM/PETHEMA (Grupo Español de Mieloma/Programa para el Estudio de la Terapéutica en Hemopatías Malignas) cooperative study group.**

| **Affiliation** | **Investigator** |
| --- | --- |
| Complejo Hospitalario Costa del Sol | María Casanova |
| Hospital Especialidades de Jerez de la Frontera | José Luís Guzman |
| Hospital Universitario Nuestra Señora de Valme | Eduardo Ríos |
| Hospital Universitario Virgen de las Nieves | Rafael Ríos |
| Complejo Hospitalario Regional Virgen del Rocío | Jesús Martín |
| Hospital Clínico Universitario Lozano Blesa | Victoria Dourdil |
| Hospital Universitario Central de Asturias | Ana Pilar González |
| Hospital Cabueñes | María Esther González |
| Hospital Universitario Son Espases | Antonia Sampol |
| Hospital Son Llátzer | Joan Bargay Lleonart |
| Hospital de Gran Canaria Dr. Negrín | Alexia Suárez |
| Hospital Universitario de Canarias | Miguel-Teodoro Hernández |
| Hospital Universitario Marqués de Valdecilla | Carmen Montes |
| Hospital General de Ciudad Real | Belén Hernández |
| Complejo Hospitalario de Toledo | Felipe Casado |
| Hospital Universitario de Guadalajara | Dunia de Miguel |
| Hospital Nuestra Señora del Prado (Talavera) | Fernando Solano |
| Hospital General de Albacete | Ángela Ibáñez |
| Hospital Clínico de Salamanca | María-Victoria Mateos  Ramón García-Sanz |
| Complejo Hospitalario H. General de Segovia | Aránzazu García-Mateo |
| Hospital de León | Fernando Escalante |
| Hospital Universitario Rio Hortega | Javier García |
| Hospital Clínico Universitario de Valladolid | Alfonso García de Coca |
| Hospital Santa Bárbara | Carlos Aguilar |
| Hospital Universitario de Burgos | Jorge Labrador |
| Hospital Althaia, Xarxa Asistencial de Manresa (Sant Joan de Deu) | Elena Cabezudo |
| Hospital Clinic, CIBERONC, Barcelona | Joan Bladé  Laura Rosiñol |
| ICO-L’Hospitalet, IDIBELL | Anna Sureda |
| ICO Girona, H. Universitario de Girona Dr. Josep Trueta | Yolanda González |
| Hospital Universitari Joan XXII de Tarragona | Lourdes Escoda |
| Hospital Universitari Arnau de Vilanova de Lleida | Antonio García |
| Hospital del Mar | Eugenia Abella |
| Hospital de Sabadell (Parc Taulí) | Joan-Alfons Soler |
| Hospital Universitario Mútua de Terrassa | Josep-María Martí |
| Hospital Universitari Germans Trias i Pujol | Albert Oriol |
| Hospital de la Santa Creu i Sant Pau | Miquel Granell |
| Hospital Vall d´Hebrón | Mercedes Gironella |
| Hospital San Pedro de Alcántara (Complejo Hospitalario de Cáceres) | Carmen Cabrera |
| Complejo Hospitalario Universitario de Santiago | Marta-Sonia González |
| Complejo Hospitalario de Pontevedra | Ana Dios |
| Complejo Hospitalario de Ourense | José-Ángel Méndez |
| Hospital San Pedro | María Josefa Nájera |
| Hospital Universitario Fundación de Alcorcón | Francisco-Javier Peñalver |
| Hospital Universitario 12 de Octubre | Juan-José Lahuerta  Joaquín Martínez-López |
| Hospital de Fuenlabrada | Pilar Bravo |
| Hospital General Universitario Gregorio Marañón | Cristina Encinas |
| Hospital Universitario Infanta Leonor | José-Ángel Hernández-Rivas |
| Hospital Universitario HM Sanchinarro | Jaime Pérez de Oteyza |
| Centro Oncológico MD Anderson | Rebeca Iglesias del Barrio |
| Hospital Universitario La Paz | Ana López de la Guía |
| Hospital Universitario de la Princesa | Adrián Alegre Amor |
| Fundación Jiménez Díaz-UTE | Elena Prieto Pareja |
| Hospital Universitario Puerta de Hierro Majadahonda | Isabel Krsnik |
| Hospital Universitario Ramón y Cajal | Maria-Jesús Blanchard |
| Hospital Clínico San Carlos | Belén Iñigo |
| Hospital Severo Ochoa | Rosalía Riaza |
| Hospital Infanta Sofía | Eugenio Giménez |
| Hospital del Tajo | Elena Ruiz |
| Hospital Morales Meseguer | Felipe de Arriba |
| Hospital Universitario Virgen de la Arrixaca | Jose-María Moraleda |
| General Universitario Santa Lucia | Marta Romera |
| Clínica Universidad de Navarra | Jesús F. San-Miguel  Paula Rodríguez-Otero |
| Complejo Hospitalario de Navarra | José María Arguiñano |
| Hospital Universitario de Cruces | María Puente |
| Hospital de Txagorritxu | Ernesto Pérez-Persona |
| Hospital Clínico Universitario de Valencia | Ana Isabel Teruel |
| Hospital Universitario Doctor Peset | Javier de la Rubia |
| Hospital Universitario La Fe | Isidro Jarque |
| Hospital General Universitario de Alicante | María Blanca Villarrubia |
| Hospital TORREVIEJA SALUD UTE | Pedro Luis Fernández |
| Hospital del Vinalopó | Pedro Luis Fernández |
| Hospital Universitario Quirónsalud Madrid | Carmen Martínez  Adrián Alegre |
| Hospital Universitario Marqués de Valdecilla, (IDIVAL) | Enrique M. Ocio |
